# Recurrence and Treatment after Renal Transplantation in Children with FSGS

**DOI:** 10.1155/2016/6832971

**Published:** 2016-04-24

**Authors:** Hee Gyung Kang, Il-Soo Ha, Hae Il Cheong

**Affiliations:** ^1^Department of Pediatrics, Seoul National University Hospital, Seoul 03080, Republic of Korea; ^2^Research Coordination Center for Rare Diseases, Seoul National University Hospital, Seoul 03080, Republic of Korea; ^3^Kidney Research Institute, Medical Research Center, Seoul National University College of Medicine, Seoul 03080, Republic of Korea

## Abstract

Focal segmental glomerulosclerosis (FSGS) is a common cause of end-stage renal disease and a common pathologic diagnosis of idiopathic nephrotic syndrome (NS), especially in steroid-resistant cases. FSGS is known to recur after kidney transplantation, frequently followed by graft loss. However, not all patients with FSGS suffer from recurrence after kidney transplantation, and genetic and secondary FSGS have a negligible risk of recurrence. Furthermore, many cases of recurrence achieve remission with the current management of recurrence (intensive plasmapheresis/immunosuppression, including rituximab), and other promising agents are being evaluated. Therefore, a pathologic diagnosis of FSGS itself should not cause postponement of allograft kidney transplantation. For patients with a high risk of recurrence who presented with classical symptoms of NS, that is, severe edema, proteinuria, and hypoalbuminemia, close monitoring of proteinuria is necessary, followed by immediate, intensive treatment for recurrence.

## 1. Introduction

Focal segmental glomerulosclerosis (FSGS) is a common cause of end-stage renal disease (ESRD). In children, the major causes of ESRD are congenital anomalies of the kidney and urinary tract and hereditary nephropathies, followed by FSGS as the most common form of acquired glomerulopathies causing ESRD [1, 2]. FSGS is the second-most common pathologic diagnosis of idiopathic nephrotic syndrome (NS) [3, 4]. Although the majority of pediatric idiopathic NS patients respond to steroid treatment, some are resistant to treatment and eventually progress to ESRD [5], and their renal pathology often reveals FSGS. Because FSGS is known to recur after kidney transplantation, frequently followed by graft loss in up to 60% of the cases [6–9], the diagnosis of idiopathic FSGS requires a thorough discussion of its prognosis with patients and their families. However, not all patients with FSGS suffer from recurrence after kidney transplantation, and many cases of recurrence achieve remission with the current management of recurrence and enjoy life as a kidney allograft recipient for as long as the average kidney recipient [10–12]. In this paper, the current knowledge of the risk factors for recurrence of FSGS and its treatment in children will be reviewed.

## 2. Who Is at Risk of Recurrence and Who Is Not?

The reported rates of recurrence are quite variable, from 6 to 58%, depending on the characteristics of the population studied [11–14]. The suggested risk factors for recurrence include the age at onset of disease [14–16], a rapid progression to ESRD (<48–72 months) [17–21], and a history of previous recurrence in an allograft [6, 18, 22]. Pathologic characteristics of the native kidney biopsy, such as mesangial hypercellularity [23] and fewer sclerotic glomeruli [19, 20], and a living donor allograft [24] have also been proposed as risk factors but have not been confirmed [25]. Native kidney nephrectomy prior to kidney transplantation has been suggested by some as a preventive measure of recurrence [21, 26, 27], but it has not been effective and has even shown a higher risk of recurrence in other reports [10, 28, 29]. According to our experiences with 38 children with FSGS, most of those with a later onset (≥6 yrs. old) and a progression to ESRD in the 24–72 months after onset of NS experienced recurrence, whereas those who had an earlier onset (<6 yrs.) of NS with a faster progression (<18 months) did not have recurrence [11]. There has been controversy over the onset age group that is at risk of recurrence; generally younger patients are considered to be at a higher risk than older patients [13], but some studies have reported no differences between adults and children [30] and even higher risks in adults than in children [25]. The main reason for these differences could be the small sample size of the study populations in most of the reports. In addition, two more aspects should be considered.

First, there are several genetic defects that cause FSGS [31–33], and the frequency and distribution of the genetic types of FSGS differ between populations. For example, the* NPHS2* mutation is the dominant cause of genetic FSGS in European countries, but it is rare in Koreans and the Japanese [31, 34–36]. Although idiopathic steroid-resistant NS (SRNS) with FSGS pathology is believed to be caused by some circulating factors [37] and is therefore prone to recur after kidney transplantation, most genetic FSGS have defective components of the kidneys, particularly podocytes, and therefore their risk of recurrence is low if not zero [16, 32, 36, 38–40]. Some genetic FSGS are characterized by an early onset of SRNS; some syndromic FSGS are accompanied by extra-renal symptoms that may not be evident at the onset of SRNS, thus mimicking idiopathic SRNS. Because a genetic diagnosis of SRNS-FSGS has not yet been incorporated as a routine component of clinical practice in most parts of the world, we do not know how many of the patients previously categorized as SRNS-FSGS have genetic FSGS. In fact, some of the cases that we previously reported as idiopathic SRNS-FSGS were recently found to have mutations in* COQ6* [41] (in patients with progressive hearing loss) or a newly found FSGS-causing gene* NUP107* [42] (unpublished data); these patients had an earlier onset (<6 yrs.) of NS with a faster progression (<18 months) and did not have recurrence [11]. A recent report by Ding et al. showed that children with SRNS who initially responded to steroid treatment were at risk of recurrence after kidney transplantation [43]. This finding may also imply that these cases have nongenetic FSGS. In other words, the wide range of risks of recurrence found in the literature seems to have been dependent on whether genetic testing was broadly performed in the studied cohort.

Second, FSGS is a pathologic diagnosis, and there are multiple causes other than idiopathic SRNS that lead to FSGS [44]. Some of the causes are evident, such as chronic infection (e.g., HIV infection) or reflux nephropathy; however, others are not. Therefore, the distinction between “secondary” and primary (idiopathic) FSGS is not always clear. Similar to genetic FSGS, FSGS secondary to other causes does not recur after kidney transplantation if the causes no longer exist after kidney transplantation; some of the reported FSGS cases without recurrence may in fact have been secondary FSGS. In our own clinical experience, patients who presented with proteinuria but without edema did not experience recurrence; although there was no identifiable cause of FSGS and they were therefore categorized as primary FSGS, we speculate that these cases may have had obscure causes leading to FSGS [11]. Therefore, the pathologic diagnosis of FSGS itself does not mean that the disease could recur after kidney transplantation.

## 3. Are There Biomarkers Predicting Recurrence?

**Figure 1:**
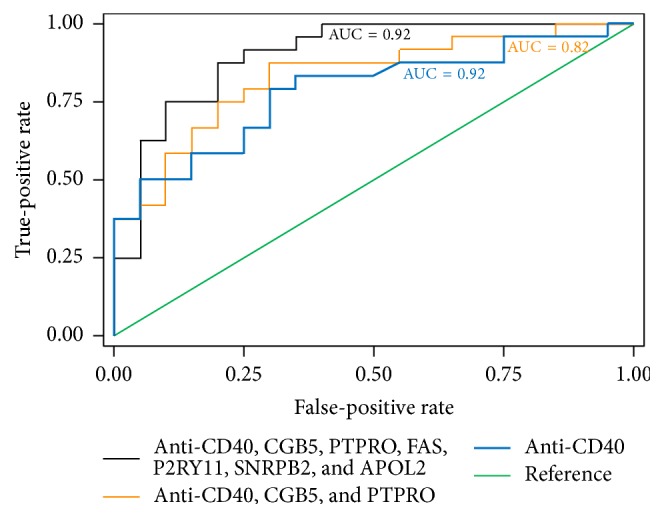
Validation of the FAST Ab panel in rFSGS and the predictive accuracy of the subsets of this panel. ROC analysis for three fitted logistic regression models. The outcome was recurrence versus nonrecurrence of FSGS, and the independent predictors were the log-transformed relative fluorescent signal values of seven Abs: CD40, PTPRO, FAS, CGB5, SNRPB2, APOL2, and P2RY11. The three logistic regression models fitted are shown. Model 1 used the FAST (FSGS antibody serological test) panel with all seven Abs, resulting in an AUC = 0.9. Model 2 used three Abs (CD40, PTPRO, and CGB5), and its ROC curve had an AUC of 0.82. Model 3 used only CD40 Ab data for the ROC analysis, resulting in an AUC of 0.77. Reproduced with permission from The American Association for the Advancement of Science © 2014 (AAAS), Delville et al. [45].

**Figure 2:**
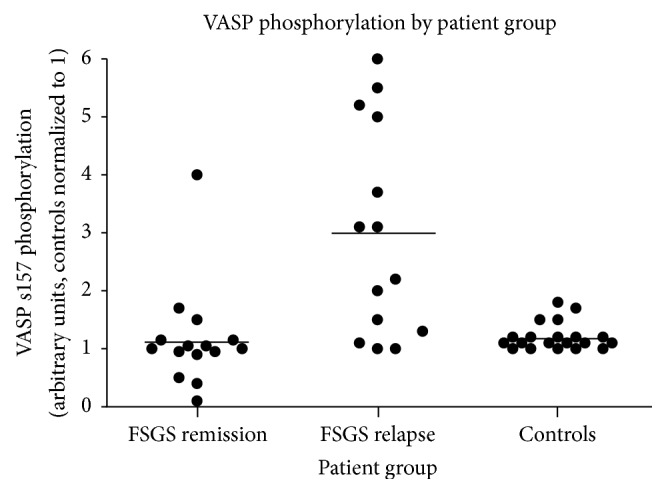
Scatter plot of VASP phosphorylation levels by patient group. Phosphorylation for each individual sample was assigned a densitometry value relative to the control (normal) plasma sample from the same gel, which was normalized to 1. Reproduced with permission from Wiley © 2012 Pathological Society of Great Britain and Ireland, Harris et al. [46].

Despite a decades-long search for circulating “permeability factor(s)” causing FSGS [47], these factors remain elusive [37]. When soluble urokinase receptor (suPAR) was reported to be a candidate permeability factor [48], this news was met with excitement. However, contradicting reports followed [49], and therefore the usefulness of suPAR as a biomarker predicting recurrence is currently doubted. Cardiotrophin-like 1 (CLC-1) is another candidate that has been proposed by Savin's group [50] and is awaiting validation. Similar to other circulating factors, autoantibodies including anti-CD40 antibody have been proposed that have shown a good predictive accuracy of recurrence (Figure 1), but these candidates require further validation in clinical trials [45]. Another approach to identifying biomarkers is the assessment of podocyte changes in response to suspicious factor(s). Vasodilator-stimulated phosphoprotein (VASP) in human podocytes has been shown to be phosphorylated in response to plasma from patients with posttransplant recurrence but not to plasma from non-FSGS, and genetic FSGS cases did not show this effect on podocytes (Figure 2). Once these promising biomarkers are validated and incorporated into clinical practice, we will be able to better predict whether recurrence will occur in a certain patient after kidney transplantation [46]. This would enable us to properly evaluate the efficacy of prophylactic management such as prophylactic plasmapheresis/immunoadsorption or immunosuppression and to possibly conduct preventive measures before transplantation [9].

## 4. How to Treat Recurrence

**Figure 3:**
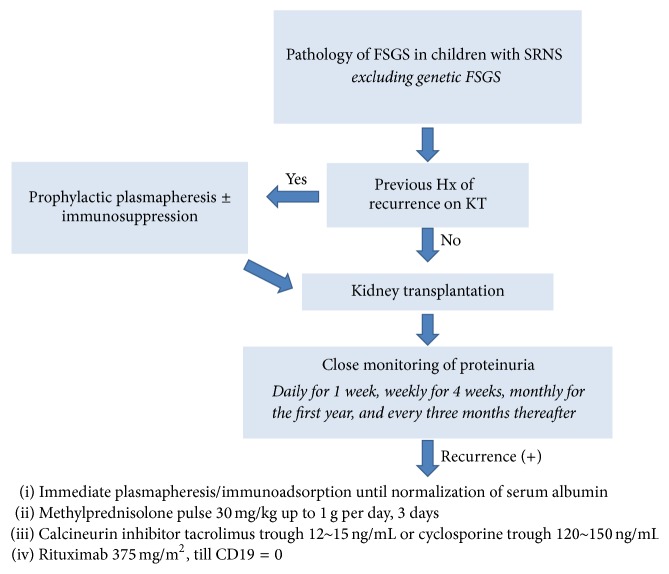
Approach for patients with SRNS-FSGS, authors' suggestion.

Although the recurrence of FSGS is a significant risk factor of graft loss [24, 29, 30, 51, 52], the outcomes of recurrent FSGS have so much improved that primary FSGS is no longer considered a contraindication of transplantation. The remission rate of pediatric recurrent FSGS has been reported to be as high as 70% [11, 12]. The mainstay of treatment for recurrent FSGS is plasmapheresis (removing 1.5 plasma volumes with 5% albumin replacement)/immunoadsorption because recurrence is believed to be caused by circulating factor(s) [53–55]. There have been no prospective randomized clinical trials to compare the efficacies of plasmapheresis and immunoadsorption, and the outcomes of studies using either method seem similar; therefore, the choice between these two methods depends on their availability and the preference of the treating physician [55–57]. It is important to begin treatment as soon as possible because removal or replacement of the FSGS-causing circulating factor(s) should be instituted before irreversible damage can be inflicted to the glomeruli [58]. While there is no evidence that anuric status at the time of transplantation prevents recurrence, recurrence can be detected more promptly if the patient had been anuric, and therefore native kidney nephrectomy can be considered in patients with residual urine output. Once the factors are removed, maintenance immunosuppression against graft rejection would also suppress the resurgence of the source of FSGS. It seems that, for some patients, plasmapheresis/immunoadsorption is sufficient to induce remission of recurrent FSGS [55, 59]. For others, intense immunosuppression with high-dose methylprednisolone, cyclosporine [12], cyclophosphamide [9], or rituximab [60] at various combinations is necessary. Although high-dose cyclosporine has been advocated as necessary by some [9, 12] and tacrolimus as a replacement for cyclosporine has been questioned [9], in our own clinical experience, tacrolimus trough levels of 12 to 15 ng/mL in combination with high-dose methylprednisolone and rituximab in addition to immediate and intense plasmapheresis have worked well, achieving remission in more than 80% of recent recurrent cases (unpublished data). The addition of rituximab to the current strategy against recurrent FSGS seems beneficial, demonstrating a response rate of up to 79% in recurrent FSGS [60, 61]. Although the optimal dosage (375 mg/m^2^ up to 6 doses or a single dose of 100 mg [62]) and mechanism of action (eradication of B lymphocytes or binding to sphingomyelin phosphodiesterase acid-like 3b [SMPDL-3b] on podocytes [63]) of this medication remain elusive, a single dose or two of 375 mg/m^2^ rituximab have been satisfactory in achieving sustained complete remission in our practice (Figure 3).

There are several promising therapeutic agents that are awaiting validation. Cytotoxic T-lymphocyte-associated antigen 4-immunoglobulin fusion protein (CTLD4-Ig, abatacept) has been tested on the basis that B7-1 (CD80) induction on podocytes plays an important role in the pathogenesis of proteinuria [64] and could eradicate proteinuria in recurrent FSGS [65]; however, the long-term efficacy of this agent has not been confirmed [66]. Of note, another form of CTLA4-Ig, belatacept, is being evaluated as a long-acting maintenance immunosuppressant against kidney allograft rejection [67]. CTLA4-Ig, a costimulatory inhibitor that competes with B7-1, could be indicated in the suppression of both allograft rejection and proteinuria. However, belatacept has been shown to increase the risk of posttransplant lymphoproliferative disease in Epstein-Barr virus- (EBV-) naïve patients [68]. Because the majority of pediatric recipients are naïve to EBV infection, caution is warranted when considering CTLA4-Ig as a therapeutic agent against recurrent FSGS in children. Another agent of interest is galactose. In the search for a “permeability factor” causing FSGS, galactose was found to bind to the factor(s) and eliminate their proteinuric effect [69, 70]. In addition to the clinical trials of this agent in pediatric SRNS [71, 72], anecdotal cases of significant improvement have been reported in recurrent FSGS [73, 74]. Successful treatment of recurrent FSGS has also been reported with anti-TNF-*α* treatment [75] (based on the upregulation of TNF-*α* mRNA in patients with FSGS [76]), adrenocorticotrophic hormone gel [77], and allogeneic mesenchymal stem cells [78, 79].

An important point is that treatment failure on native kidneys does not predict treatment failure for posttransplantation recurrence of FSGS. Why? For recurrent FSGS after transplantation, we start treatment almost immediately, before the formation of sclerosis. This implies that delays in treating the native kidney lead to treatment failure, resulting in progressive renal damage. Therefore, if we are equipped with reliable biomarkers that indicate which medications will be effective in specific patients at the time of NS diagnosis, we will be able to tailor the treatment of pediatric NS, thus applying “precision medicine” to these patients.

## 5. How to Prevent Recurrence

While any discussion of preventive treatments efficacy is futile because we do not know whether subjects will experience recurrence after transplantation, patients who had lost their previous allograft to recurrence of FSGS have particularly high risk of recurrence [6, 18, 22]. For these patients, to eliminate circulating factors, preemptive plasmapheresis/immunoadsorption is considered, three to five sessions prior to the transplantation followed by immediate posttransplant sessions of three to five [80–85]. Additional single dose of rituximab (375 mg/m^2^) along with immunosuppression of corticosteroid, calcineurin inhibitor, and mycophenolate mofetil for two weeks prior to kidney transplantation was shown to prevent recurrence [81, 84].

## 6. Conclusion

Recurrence after kidney transplantation is devastating for patients and families. However, the outcomes of recurrent FSGS are quite good with the current management strategy; therefore, the pathologic diagnosis of FSGS itself should not be a cause for postponing allograft kidney transplantation. For patients with a high risk of recurrence, close monitoring of proteinuria as suggested by the Kidney Disease: Improving Global Outcomes guidelines for the management of kidney transplant recipients [86] is necessary, followed by immediate, intensive treatment for recurrence, as suggested in Figure 3.
